# Functional electrical stimulation cycling exercise after spinal cord injury: a systematic review of health and fitness-related outcomes

**DOI:** 10.1186/s12984-021-00882-8

**Published:** 2021-06-12

**Authors:** Jan W. van der Scheer, Victoria L. Goosey-Tolfrey, Sydney E. Valentino, Glen M. Davis, Chester H. Ho

**Affiliations:** 1 Peter Harrison Centre for Disability Sport, School for Sport, Exercise and Health Sciences, Loughborough University, Epinal Way, Loughborough, LE11 3TU UK; 2 The Healthcare Improvement Studies (THIS) Institute, Department of Public Health and Primary Care, School of Clinical Medicine, University of Cambridge, Cambridge Biomedical Campus, Clifford Allbutt Building, Cambridge, CB2 OAH UK; 3 Department of Kinesiology, McMaster University, Room IWC EG115, 1280 Main St. W., Hamilton, ON L8S 4K1 Canada; 4 Discipline of Exercise and Sport Sciences, Faculty of Medicine and Health, Sydney School of Health Sciences, University of Sydney, Sydney, NSW 2006 Australia; 5 Division of Physical Medicine & Rehabilitation, Faculty of Medicine & Dentistry, University of Alberta, Edmonton, AB T6G 2R3 Canada

**Keywords:** Spinal cord injury, Functional electrical stimulation, Exercise, Systematic review, Clinical practice guidelines

## Abstract

**Objectives:**

The objective of this review was to summarize and appraise evidence on functional electrical stimulation (FES) cycling exercise after spinal cord injury (SCI), in order to inform the development of evidence-based clinical practice guidelines.

**Methods:**

PubMed, the Cochrane Central Register of Controlled Trials, EMBASE, SPORTDiscus, and CINAHL were searched up to April 2021 to identify FES cycling exercise intervention studies including adults with SCI. In order to capture the widest array of evidence available, any outcome measure employed in such studies was considered eligible. Two independent reviewers conducted study eligibility screening, data extraction, and quality appraisal using Cochranes’ Risk of Bias or Downs and Black tools. Each study was designated as a Level 1, 2, 3 or 4 study, dependent on study design and quality appraisal scores. The certainty of the evidence for each outcome was assessed using GRADE ratings (‘High’, ‘Moderate’, ‘Low’, or ‘Very low’).

**Results:**

Ninety-two studies met the eligibility criteria, comprising 999 adults with SCI representing all age, sex, time since injury, lesion level and lesion completeness strata. For muscle health (e.g., muscle mass, fiber type composition), significant improvements were found in 3 out of 4 Level 1–2 studies, and 27 out of 32 Level 3–4 studies (GRADE rating: ‘High’). Although lacking Level 1–2 studies, significant improvements were also found in nearly all of 35 Level 3–4 studies on power output and aerobic fitness (e.g., peak power and oxygen uptake during an FES cycling test) (GRADE ratings: ‘Low’).

**Conclusion:**

Current evidence indicates that FES cycling exercise improves lower-body muscle health of adults with SCI, and may increase power output and aerobic fitness. The evidence summarized and appraised in this review can inform the development of the first international, evidence-based clinical practice guidelines for the use of FES cycling exercise in clinical and community settings of adults with SCI.

*Registration review protocol*: CRD42018108940 (PROSPERO)

## Background

Functional electrical stimulation (FES) applies low-level electrical pulses to paretic or paralyzed muscles to restore or improve their functional capacity. It is a neuroprosthetic, therapeutic or exercise modality for individuals with a nervous system injury to reactivate the peripheral nervous system without significant lower motor neuron damage [1]. In clinical and community settings, one of the most commonly available and researched FES exercise modalities is FES-evoked cycling [2–4]. FES cycling allows people with little or no voluntary leg movement to pedal an exercise bicycle, usually indoors on a stationary system. Computer generated, low-level electrical pulses are transmitted through transcutaneous electrodes to the leg muscles. This evokes coordinated contractions and a pedaling motion that mimics voluntary exercise training. Potential or anecdotal benefits include improvements in muscle, bone and cardiovascular health, fitness, feelings of well-being, and motor function of people with neurological conditions such as stroke, multiple sclerosis and spinal cord injury (SCI) [1, 5–8].

Despite the potential and its availability, FES cycling is currently not consistently deployed as a component of the lifelong rehabilitation care plan for all eligible individuals with SCI who are responsive to FES. More evidence-based exercise and rehabilitation options would be of particular benefit to the SCI community [9], given their high risk of secondary health complications [10], and barriers to participate in exercise [11]. The availability of evidence-based clinical practice guidelines can enhance the use of therapeutic exercise and rehabilitation options [12–14]. Essential to the development of such guidelines is a systematic literature review in accordance with Grading of Recommendations Assessment, Development and Evaluation (GRADE) [13, 15, 16]. Although recent systematic reviews have provided helpful insight into specific outcomes [17–19], a comprehensive systematic review including GRADE assessments is currently not available for FES cycling research in SCI.

Accordingly, this review sought to summarize and appraise evidence of randomized controlled trials (RCTs), non-RCTs, pre-post studies, case series, case studies and cross-sectional controlled studies evaluating the effects of FES cycling exercise among adults with SCI. Any health or fitness-related outcome measures used in those studies were considered eligible for inclusion, to ensure a complete overview of what outcomes have been used in FES cycling exercise research for the SCI population. Although not a primary objective, the review also sought to provide an overview of adverse events reported in the included studies.

## Methods

We designed the review’s protocol in accordance with international reporting standards [20, 21], and in consideration for the future development of practice guidelines for clinical and community settings [14]. The review was registered in PROSPERO (CRD42018108940). Information required for compliance with the reporting standards that has not been provided in this paper can be found in an online data repository at https://osf.io/u9mvx/, including the reference list of eligible studies, a ‘grey’ literature search, data extractions and risk of bias (quality appraisal) scoring.

### Search strategy

**Fig. 1:**
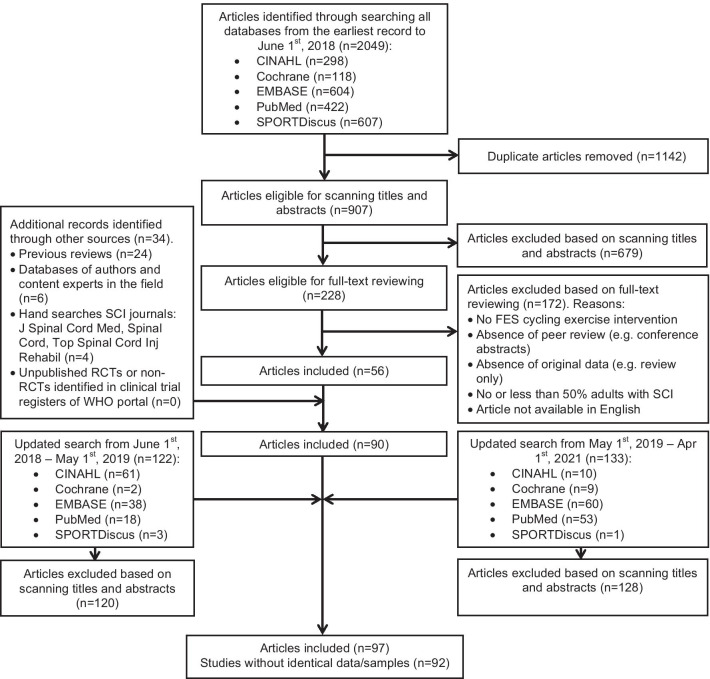
Flow chart of the literature search and selection of eligible articles. *Note* The reference list of the 97 included articles with the 92 unique datasets is provided in the online repository (https://osf.io/u9mvx/). *FES* functional electrical stimulation; *RCT* randomized controlled trial; *SCI* spinal cord injury; *WHO* World Health Organization

PubMed, the Cochrane Central Register of Controlled Trials, EMBASE (OVID), SPORTDiscus (EBSCOhost), and CINAHL (EBSCOhost) were searched from the earliest record until April 1st, 2021. To coincide with two guideline development meetings, these databases were first searched to June 2018, and then updated to May 2019 (Fig. 1). An updated search was also conducted in April 2021 (Fig. 1). An independent librarian contributed to the search strategy. Keywords were a combination of terms representing SCI (e.g., paraplegia, tetraplegia) [2], FES (e.g., functional electric stimulation, electrotherapy) and cycling (e.g., cycle, pedalling), including database-specific indexing terms (e.g., Emtree for EMBASE). The online repository (https://osf.io/u9mvx/) provides the tailored search strings for each database. To identify other relevant studies, we consulted content experts and searched the reference lists of previous reviews (Fig. 1). To identify potential publication bias, the World Health Organization trial registry was searched for unpublished RCTs or non-RCTs matching the study eligibility criteria (i.e., ‘grey’ literature search). Language familiarity of the review team limited the search to peer-reviewed articles written in English, which we anticipated to have limited effect on our conclusions [22].

### Study eligibility criteria

As part of the guideline development process, international stakeholder meetings with FES users, researchers, clinicians and other practitioners were conducted in 2018 and 2019 (Edmonton, Canada; Loughborough, UK; manuscript in preparation). These meetings were informed by a 2016 overview of SCI exercise evidence that included FES studies [2], and an additional scoping review on FES exercise RCTs and non-RCTs. The preliminary available evidence and stakeholders discussions informed the decision to focus the guideline development process (including the current review) on FES cycling, given that it is one of the most commonly used and accessible FES modalities with the largest body of high-quality evidence supporting it. Informed by these stakeholder meetings, the following selection of study eligibility criteria was established:

- *Participants*: Studies that included a sample of at least 50% with adults (≥ 16 years) with traumatic or non-traumatic SCI (any time post-onset SCI) who were eligible and responsive to FES cycling. Excluded were those with a congenital condition (e.g., spina bifida), or a progressive disease (e.g., multiple sclerosis with spinal cord involvement).
- *Interventions*: Studies that employed an FES lower-body cycling exercise intervention and describing exercise prescription parameters such as intervention period (e.g., 12 weeks), exercise frequency (e.g., three times per week), and/or exercise duration (e.g., 30 min per session). FES cycling was defined as a modality whereby transcutaneous electrical currents are applied to paralyzed or paretic muscles, with the necessary stimulation characteristics provided to evoke muscle contractions for lower-body cycling movements. “Exercise” was defined as planned, structured, and repetitive physical activity that is performed to improve or maintain physical fitness component(s) [23]. Excluded were interventions shorter than two weeks [2], and interventions that did not allow inferences about the specific contributions of FES cycling, e.g. activity-based restorative therapy [24, 25].
- *Comparator/Control*: Studies were eligible as a controlled study if the comparator for the exercise intervention was a control group not receiving an FES cycling exercise intervention. Receiving usual care (e.g., during the inpatient rehabilitation period) was also accepted as a control condition when the exercise group also received this usual care in addition to the exercise intervention [2]. Studies comparing two FES cycling interventions (e.g., low-cadence vs high-cadence cycling) were included and appraised as pre-post studies.
- *Outcome measures*: Rather than focusing on a fixed set of outcomes, studies employing any type of health or fitness-related outcome measure were included, so long as they were measured in response to FES cycling exercise in participants with SCI. This wide array was chosen to ensure a complete overview of what outcomes have been used in FES cycling intervention research for the SCI population.
- *Study designs*: RCTs, non-RCTs, pre-post, case series, case report, and cross-sectional controlled studies, in order to capture all available evidence beyond the limited available exercise RCTs in SCI [2]. Only cross-sectional studies without a control group were excluded, given the impossibility to make any assumptions about causality.

### Study eligibility screening

Co-author SEV and a review team with content expertise (see ‘Acknowledgements’) conducted the study selection, supervised by primary author JWvdS. Two reviewers screened the titles and abstracts independently after duplication removal. Full-text articles were retrieved if one or both reviewers considered a study potentially eligible for inclusion. Two reviewers independently reviewed the full-text articles for eligibility, while recording all reasons for exclusion. Any disagreements during this process were discussed between the reviewers. If no consensus was reached, JWvdS adjudicated the inclusion/exclusion of an article. Reviewers were not blinded to authors or journals.

### Data extraction

The data extraction sheets are provided in https://osf.io/u9mvx/. Data extracted included details on: study design, demographics, spinal cord lesion characteristics, training status at baseline, participant exclusion criteria, intervention location and environment, exercise prescription, neuromuscular stimulation characteristics, outcome measures, confidence intervals, statistical power, and adverse events. JWvdS and SEV pilot tested preliminary data extractions sheets and developed the final data extraction sheets with the other authors. Using these, two reviewers independently extracted data from a sample of eligible studies (10%) and achieved good agreement (at least 80% concordance), with the remainder extracted by one reviewer. JWvdS verified all data extractions.

### Risk of bias in individual studies

Two reviewers independently appraised the included RCTs using Cochrane’s RoB 2.0 [26], non-RCTs using ROBINS-I [27], and used a modified Downs and Black tool [28, 29] for the other study designs (see https://osf.io/u9mvx/. The reviewers discussed differences until full consensus was reached, if necessary adjudicated by JWvdS. One of 4 Levels of evidence was established for each study (Table 1), based on the strength of the study design and cut-off scores from the quality appraisal tools, similar to previous approaches [2, 29]. A Level 1 study indicated a study with the least risk of bias, and a Level 4 study highest risk of bias.

**Table 1:** Rating system for risk of bias of individual studies

Level 1	∙ Randomised controlled trials with Low risk of bias as assessed by RoB 2.0
Level 2	∙ Randomised controlled trials with Some concerns or High risk of bias as assessed by RoB 2.0 ∙ Non-randomised controlled trials (including case–control, prospective and retrospective cohort studies) with Low or Moderate risk of bias as assessed by ROBINS-I
Level 3	∙ Non-randomised controlled trials (including case–control, prospective and retrospective cohort studies) with Serious or Critical risk of bias as assessed by ROBINS-I ∙ Pre-post studies (any Downs and Black score)
Level 4	∙ Case series defined by 3–5 individuals only (any Downs and Black score) ∙ Case report defined by 1–2 individuals only (any Downs and Black score) ∙ Cross-sectional controlled study (any Downs and Black score)

RCTs assessed with Cochrane’s Risk of Bias (RoB) 2.0 [21], non-RCTs with Cochrane’s The Risk Of Bias In Non-randomized Studies—of Interventions (ROBINS-I) tool [22], and all other study designs using a modified version of the Downs and Black scale which ranges from 0 to 28 points [23, 24]. Further details on risk of bias assessment: https://osf.io/u9mvx/

### Evidence summary

The outcome measures that were identified during data extraction were categorized, in accordance with a previously published systematic review on cardiorespiratory fitness, power output, muscle strength, cardiometabolic health and bone health [2]. We then expanded the outcome categories to fit the wider scope of outcomes covered by this review (e.g., subjective well-being [30]) and this review’s specific focus on FES cycling exercise. The proposed categorisation of outcome measures was validated and confirmed by FES content experts as part of the international expert panel meetings to develop FES cycling guidelines. The following outcome categories were defined:

- Muscle health: Including measures representing muscle volume, circumference and fiber type composition (e.g., cross-sectional leg muscle area, mid-thigh muscle volume, % type IIa vs type IIb fibers)
- Power output: Including measures representing lower-body power output (e.g., peak power output during an incremental FES cycle test, or average power output during training)
- Aerobic fitness: Including measures representing peak oxygen uptake and respiratory capacity (e.g., peak oxygen uptake during an incremental FES cycle test, tidal volume)
- Muscle strength: Including measures representing isometric or isokinetic muscle force and torque (e.g., electrically stimulated peak leg extension torque, isometric knee extension force)
- Fat mass: Including measures representing adipose tissue (e.g., abdominal ectopic fat, cross-sectional leg fat area)
- Cardiovascular and metabolic factors: Including measures representing cardiac, arterial and metabolic structure and function (e.g., arterial pulse wave velocity, insulin sensitivity, cytokine profiles)
- Bone health: Including measures representing bone mineral density (BMD), bone turnover markers and histomorphometry (e.g., whole-body BMD, bone-specific alkaline phosphatase, N-telopeptides)
- Subjective well-being: Including measures representing anxiety and depression, life satisfaction, perceived stress (e.g., Hospital Anxiety and Depression Scale, World Health Organization Quality of Life Scale, Perceived Stress Scale)
- Functional and neurological outcomes: Including measures representing functional independence or neurological recovery (e.g., motor and sensory function, 6-min walking test, Functional Independence Measure, Spinal Cord Independence Measure)
- Other secondary health conditions: Including measures representing SCI-specific secondary conditions such as spasticity, bowel function or oedema (e.g., Modified Ashworth Scale, Neurogenic Bowel Dysfunction Score).

Following, the review team designated for each study whether the intervention showed an improvement in an outcome category or not, similar to a previous review [2]. Given the lack of benchmarks for clinically meaningful improvements [31], and the anticipated large variety of outcome measures [2], “improvement” was defined as a statistically significant positive change following the intervention in at least one of the outcome measures within an outcome category [2]. For studies in which statistics were not applied, for example in a case series study, when all participants improved in an outcome, this was classified as an improvement. A study’s intervention could also be designated to provide an “inconclusive” result, for example when one subgroup improved in contrast to another, when one measure indicated an improvement and another measure of that same outcome category indicated worsening, or when no statistics were provided in a pre-post study. JWvdS verified all designations.

Studies showing an improvement or not were summarized separately for Level 1, 2, 3 and 4 studies across each outcome category, to enable the evidence appraisal using GRADE (see below). Given the variety of study designs, interventions and reported outcome measures, we did not consider it feasible or valid to synthesise the results quantitatively using meta-analyses or forest plots. Combining data on these measures for the purpose of meta-analysis could be misleading if the magnitude of effects differed across outcomes and study designs. The potential for meta-analyses and forest plots was also limited by the low reporting quality in many studies. For example, some studies failed to provide group descriptive statistics, while many studies did not report effect sizes or relative differences within and between groups.

### Evidence appraisal using GRADE

GRADE methodology was used to assess certainty of the evidence for each outcome category [13, 15]. The GRADE method prescribes assessing the body of evidence (i.e., all studies taken together) for the following criteria: *very serious risk of bias, serious risk of bias, inconsistency*, *imprecision*, *indirectness*, and *publication bias* (Table 2) [13, 15]. If one or more of those issues appear, GRADE certainty in the evidence is to be downgraded from ‘High’ to ‘Moderate’, ‘Low’ or ‘Very Low’ [13, 15]. Conversely, the GRADE method prescribed that certainty in the evidence can be upgraded if there are indications of a *dose–response gradient*, *plausible bias* or *large magnitude of effects* in lower-quality studies [13, 15]. The higher the certainty, the more confidence one can have that the measured effect aligns with the true effect [16]. ‘Low’ or ‘Very Low’ certainty in the evidence does not imply an intervention does not work; it merely indicates that confidence is limited about the measured effect aligning with the true effect [16].

**Table 2:** Criteria and benchmarks to assess certainty of the evidence using GRADE [10, 12, 16]

GRADE criterion	Meaning	Benchmark used in this review
Risk of bias	Quality of the evidence	No *risk of bias* if at least one Level 1 study was present *Serious risk of bias* if only one Level 2 was present *Very serious risk of bias* if no Level 1 or 2 studies were present
Inconsistency	Results for a given outcome not similar across studies	No *inconsistency* if improvements shown in at least: – Two thirds of Level 1 or 2 studies and half of Level 3 or 4 studies; or – Half of Level 1 or 2 studies and two third of Level 3 or 4 studies; or – Two thirds of Level 3 or 4 studies in absence of Level 1 or 2 studies
Imprecision	Insufficient statistical power or wide confidence intervals	No *imprecision* if at least one study was sufficiently powered and at least one study showed narrow confidence intervals surrounding the estimate of effects
Indirectness	Evidence differs from study eligibility criteria (PICO)	No *indirectness* if—across the studies—the following participant characteristics were represented: male/female, young and middle-aged adults (16–65 years) and older adults (> 65 years), time since injury > 1 year and > 1 year, and lesion characteristics (AIS and lesion level) with sufficient lower motor neuron capacity to respond to FES cycling
Publication bias	Selective publication of studies	*Publication bias* present if unpublished studies added to the evidence summary would have changed assessment of any of the criteria shown above
Reasons for upgrading level of certainty in the evidence	If lower-quality studies provide convincing evidence	– *Consistent effects* across a large number of Level 2, 3 or 4 studies – *Plausible bias* caused by including participants not responsive to FES cycling – *Dose–response gradient* present in one study or across all studies

GRADE certainty in the evidence can be ‘High’, ‘Moderate’, ‘Low’ or ‘Very Low’, subject to the presence of the criteria presented in this table [10, 12]

*AIS* American Spinal Injury Association Impairment Scale, *PICO* Participants, Intervention, Comparator, Outcomes

For the purpose of this review, we developed benchmarks for each GRADE criterion (Table 2) in accordance with previously developed criteria [2]. GRADE certainty in the evidence was downgraded by two levels (e.g., from ‘High’ to ‘Low’) if there was *very serious risk of bias*. It was downgraded by one level (e.g., from ‘High’ to ‘Moderate’) when *serious risk of bias, inconsistency, imprecision, indirectness* or *publication bias* was present. Certainty of the evidence was upgraded by one level if we observed *consistent effects, plausible bias* and/or a *dose–response gradient* across the Level 2, 3 and 4 studies.

### Adverse events

Although not a primary objective of this review, the included studies were summarized for their descriptions of *suspected adverse reactions*. These were defined in accordance with the US FDA as adverse events for which there was a reasonable possibility that the FES intervention caused the adverse event [32]. For the studies that described adverse events, the summaries included the total number of participants reporting *serious suspected adverse reactions* (e.g., life-threatening event, event that required prolonged hospitalization), or *other suspected adverse reactions* [32].

## Results

The search strategy and eligibility screening led to the inclusion of 97 articles that comprised 92 studies without identical data/samples [33–129] (Fig. 1). The online repository (https://osf.io/u9mvx/) provides the reference list of the 97 articles, data extractions for each of the 92 studies, and details of the literature search in the trial registers. Tables 3, 4 and 5 provide an overview of extracted characteristics of participants, interventions and outcome measures.

**Table 3:** Summary of participant characteristics across all studies

Demographics	Total participants: 999 Total men/women/NR: 782/143/74 Mean age reported: 36 ± 8 (20–60) years Min age reported: 27 ± 9 (16–60) years Max age reported: 47 ± 10 (20–80) years Mean TSI reported: 9.0 ± 6.7 (0.04–33) years Min TSI reported: 4.0 ± 6.6 (0.03–33) years Max TSI reported: 17.6 ± 12.6 (0.04–53) years
Lesion characteristics	Lesion level averaged* (range): C6-T8 (C1-L1) AIS A: 47 out of 92 studies AIS B: 30 out of 92 studies AIS C: 17 out of 92 studies AIS D: 6 out of 92 studies AIS NR: 30 out of 92 studies
Training status at baseline	No training in FES cycling: 66 out of 92 studies Trained in FES cycling: 8 out of 92 studies Training status NR: 18 out of 92 studies
Most frequent exclusion criteria	Bone fractures in the trochanter or pelvic area: 22 studies Presence of severe osteoporosis or similar conditions: 21 studies Too limited range of motion of hip or knee joints: 20 studies Not able to cycle due to spasticity: 17 studies Presence of pressure injuries: 16 studies

Further details on data extraction for each study: https://osf.io/u9mvx/

*AIS* American Spinal Injury Association Impairment Scale, *NR* not reported

*Averaged range calculated using coding for each lesion level

**Table 4:** Summary of intervention characteristics across all studies

Exercise prescription*	Period: 16 (8–26) weeks Frequency: 3 (2–5) times/week Duration: 30 (10–60) min/session Min cycle speed: 35 (10–50) RPM Max cycle speed: 50 (35–60) RPM
Neuromuscular stimulation characteristics*	Pulse width: 300 (200–500) µs Amplitude: 140 (0–180) mA Stimulation frequency: 35 (20–60) Hz
Intervention environment	Research centre: 24 out of 92 studies** Clinical centre: 19 out of 92 studies** Home-based: 18 out of 92 studies Environment NR: 27 out of 92 studies
Most frequent study locations	USA: 44 out of 92 studies UK: 9 out of 92 studies Australia: 7 out of 92 studies Canada: 6 out of 92 studies Denmark: 5 out of 92 studies The Netherlands: 5 out of 92 studies

Further details on data extraction for each study: https://osf.io/u9mvx/

*NR* not reported, *RPM* revolutions per minute

*Period reported as Median (interquartile range). All other values reported as Mode (range). Extreme outliers of these parameters were excluded from this summary, i.e. periods of 37 months, 56 months or 0.4–7 years; frequency of seven times/week; duration of 100 min; max cycle speed of 20 RPM

**4 studies took place in both research and clinical environments

**Table 5:** The number of Level 1, 2, 3 or 4 studies showing significant improvements for each outcome category (ordered from most to least frequently studied)

Outcome category	Total	Level 1	Level 2	Level 3	Level 4
Muscle health	30 out of 36	0 out of 1	3 out of 3	12 out of 16	15 out of 16
Power output	34 out of 35	0 out of 0	0 out of 0	29 out of 30	5 out of 5
Aerobic fitness	20 out of 26	0 out of 0	0 out of 0	17 out of 21	3 out of 5
Bone health	11 out of 23	0 out of 0	1 out of 2	6 out of 12	4 out of 9
Cardiovascular and metabolic factors	16 out of 21	0 out of 0	0 out of 0	12 out of 17	4 out of 4
Fat mass	8 out of 16	0 out of 1	1 out of 2	1 out of 3	6 out of 10
Muscle strength	12 out of 14	0 out of 0	0 out of 1	10 out of 11	2 out of 2
Other secondary health conditions	7 out of 13	0 out of 1	1 out of 2	5 out of 7	1 out of 3
Subjective well-being	7 out of 10	0 out of 1	0 out of 0	3 out of 4	4 out of 5
Functional and neurological outcomes	3 out of 5	0 out of 0	0 out of 0	3 out of 5	0 out of 0

Further details on data extraction for each study: https://osf.io/u9mvx/

*AIS* American Spinal Injury Association Impairment Scale

### Risk of bias in individual studies

Each of the 92 studies was classified for its individual Level of evidence in accordance with Table 1. Two were classified as Level 1 studies, 7 as Level 2 studies, 65 as Level 3 studies, and 18 as Level 4 studies. An RCT design was used in five studies, with RoB 2.0 scores ranging from Low to Serious risk of bias. A non-RCT design was used in four studies, with ROBINS-I scores ranging from Low to Moderate risk of bias. Downs and Black scores ranged from 4 to 22 (mean ± SD: 12 ± 4) across the studies with pre-post, case series, case report and cross-sectional designs. Detailed risk of bias scores for each checklist item of the studies are available at https://osf.io/u9mvx/.

### Participant characteristics

Overall, the evidence included 999 participants representing all demographic and spinal cord lesion characteristic strata (Table 3). Underrepresented in the evidence were women, adults > 65 years, participants with motor incomplete injuries and those with high cervical or lumbar lesions. Most participants were untrained in FES cycling exercise at baseline, although some received FES strength training before starting the intervention. They were free of bone fractures, pressure injuries or other common reasons for exclusion from participating in FES exercise.

### Intervention and control characteristics

The average intervention period across all studies was 16 weeks, mostly cycling three times per week for 30 min at 35–50 revolutions per minute, using a neuromuscular stimulation amplitude up to 140 mA, a pulse width of 300 µs, and a pulse frequency of 35 Hz (Table 4). If a form of progression was used and reported in the studies, it consisted of increasing absolute resistance or torque levels within or across sessions, based on participants’ cycling frequency, fatigue, and/or personal tolerability. None of the studies reported gauging exercise intensity using physiological criteria such as percent peak oxygen uptake or heart rate, except for peak power output.

The majority of interventions took place in research and/or clinical environments, while 19 studies employed home-based environments (Table 4). In 19 studies, FES cycling was preceded or complemented by other lower-body strength exercise, such as a number of weeks of FES quadriceps strengthening preceding subsequent weeks of FES cycling. Almost half of the studies (44 out of 92) were conducted in the USA, while 4–8 studies took place in Australia, Canada, Denmark, the Netherlands, Switzerland, or the UK (Table 4). The remainder of the studies took place in other countries across Asia, Australia, Europe, the Middle East and South America. Control groups followed usual in-patient rehabilitation care, conducted passive cycling or upper-body exercise, or did not participate in any exercise intervention.

### Outcomes

As summarized in Table 5, the most frequently employed outcome measures were indices of muscle health (e.g., muscle cross-sectional area, ratio between muscle fiber types), power output (e.g., peak power output on an incremental FES cycling test, average power output during training), or aerobic fitness (e.g., peak oxygen uptake on an incremental FES cycling test, tidal volume). For muscle health (36 studies), the one Level 1 study reported non-significant findings, while the four Level 2 studies and over 80% of Level 3 or 4 studies demonstrated significant improvements. For power output and aerobic fitness, Level 1 or 2 studies were lacking, but over 35 Level 3 and 4 studies were available. Nearly all of these studies showed significant improvements, for example in 29 out of 30 Level 3 studies on power output and 17 out of 21 Level 3 studies on aerobic fitness. Lower consistency or less evidence was available for the other outcomes (Table 5). For example, less than half of the studies on bone health (11 out of 23 studies) found significant improvements after 8–26 weeks of FES cycling exercise in measures such as bone mineral density and bone turnover markers. Studies on functional and neurological outcomes (e.g., independence measures, ISNCSCI motor scores) were limited to five Level 3 studies, of which three demonstrated significant improvements.

### Evidence appraisal using GRADE

For muscle health, the GRADE assessment identified potential *imprecision* (Table 2), due to limited or no information on statistical power or confidence intervals around effect estimates. The GRADE assessment also revealed *indirectness* (i.e., limited generalizability), but only for older adults > 65 years. The evidence on muscle health included participants with paraplegia or tetraplegia (C1 to L1, AIS A, B, C or D), 0.04–53 years post-injury (mean: 10 years), aged 16–67 years (mean: 36 years). We upgraded certainty in the evidence for muscle health by one level due to the *consistent effects* found across the large number of Level 2, 3 and 4 studies (Table 5). This led to ‘Moderate’ certainty in the evidence for any adult with SCI, and ‘High’ certainty in the evidence for young to middle-aged adults with SCI.

For power output, the GRADE assessment revealed *very serious risk of bias* due to the absence of Level 1 or 2 studies, and potential *imprecision* due to lack of information about statistical power and confidence intervals. The evidence on power output included participants with paraplegia or tetraplegia (C3 to L1, AIS A, B, C or D), 0.16–53 years post-injury (mean: 10 years), aged 17–80 years (mean: 38 years). We upgraded certainty in the evidence by one level due to the highly *consistent effects* found across the large number of Level 3 studies (Table 5). Therefore, GRADE certainty in the evidence for augmented power output was ‘Low’ for any adult with SCI.

The GRADE assessment for aerobic fitness was similar to that of power output; *very serious risk of bias* and potential *imprecision*, and strengthening of confidence in the evidence by the *consistent effects* across the Level 3 studies (Table 5). The evidence on power output included participants with paraplegia or tetraplegia (C3 to L2, AIS A, B, C or D), 0.08–33 years post-injury (mean: 9 years), aged 16–70 years (mean: 35 years). Accordingly, the GRADE assessment established ‘Low’ certainty in the evidence for improved aerobic fitness after FES cycling exercise.

The GRADE assessments led to ‘Very Low’ certainty in the evidence for the other outcomes shown in Table 5, due to an absence of Level 1 or 2 studies, effects being inconsistent across the studies, *imprecision*, and/or *indirectness*.

### Adverse events

None of the studies had adverse events as its primary outcome. Adverse events were described in 21 studies comprising 203 participants, as detailed in the data extraction table (https://osf.io/u9mvx/). Of these, 18 participants experienced *suspected adverse reactions* to FES cycling. One out of these 18 participants experienced a *serious suspected adverse reaction*; the participant was reported to be withdrawn from an FES-cycling intervention related to haemotoma development in the ischial region, which may or may not have been associated with the intervention. Seventeen participants experienced *other suspected adverse reactions* such as temporary post-exercise hypotension (n = 4), increased spasticity (n = 4), light-headedness (n = 2), skin redness (n = 2), bowl accident (n = 1), autonomic dysreflexia caused by stimulation (n = 2), increased leg swelling (n = 1), and a small quadriceps haemotoma that was resolved within 2 weeks (n = 1). Two of these could not finish the FES intervention due to increased spasticity.

## Discussion

This review has provided the first summary and appraisal of evidence for the effects of FES cycling exercise interventions on health and fitness-related outcomes measured after SCI. The GRADE assessments revealed ‘High’ certainty in the evidence for significant improvements in lower-body muscle health (e.g., larger muscle volume, shift to more fatigue-resistant fiber types), and ‘Low’ certainty in the evidence for significant improvements in power output and aerobic fitness (e.g., peak power output and oxygen uptake during an incremental FES cycling test) of adults with SCI. This review also highlighted that future high-quality research is necessary to validate conclusions about other potential benefits, such as improved cardiovascular health, and functional or neurological adaptations. The limited available evidence on adverse events suggested that harmful reactions are unlikely to occur when adults with SCI engage in FES cycling.

All but one RCT and a large number of Level 3–4 studies found significant improvements in outcomes for muscle health. The one RCT without significant improvements may be explained by a relatively short intervention duration (i.e., < 3 months), and insensitivity of its outcome measure related to the location of measurement of cross-sectional area [71, 130]. Overall, the evidence indicated that FES cycling could help counteract the vast loss of muscle mass after SCI, which can be as high as 80% when compared to able-bodied controls [131, 132]. This might reduce risk of pressure injuries [133], increase the low resting metabolic rates that can contribute to obesity [134], and enhance satisfaction with body appearance [135]. The changes in fiber type composition shown by the evidence (e.g., shift from type IIb and IIx fibers to type IIa fibers) indicate that FES cycling can help reverse the loss of oxidative capacity of paralyzed muscles [136]. This may aid beneficial vascular adaptations [137], improve aerobic metabolism [5], and reduce the onset of fatigue during further FES training [138].

A large number of Level 3 and 4 studies provided consistent evidence that FES cycling exercise could improve lower-body power output and aerobic fitness. If these improvements relate at least to some extent to the cardiovascular and cognitive health benefits found in lower-body exercise in the able-bodied population [139, 140], then FES cycling has great potential for reducing the high risk of cardiovascular and cerebrovascular conditions after SCI [141–144].

### Strengths and limitations of this review

One of the strengths of this review was the transparent use of GRADE to appraise the body of evidence for each outcome, in accordance with international standards [13, 16]. However, we also acknowledge that a sole focus on GRADE criteria may not provide recommendations that clinicians can easily utilize [145]. For example, the quality of SCI evidence about exercise will always be prone to downgrading using the GRADE criteria due to imprecision and indirectness, considering the inherent challenges in undertaking high-quality exercise research in this population [146]. These include the small potential participant pools, an inherent age and sex distribution in the SCI population traditionally representing relatively fewer women and older adults, neurological heterogeneity common in SCI samples, and the complexity of spinal cord lesion characteristics influencing outcomes. Notwithstanding, evidence-based guidelines can still be developed even when the GRADE assessment reveals ‘Low’ certainty in the evidence, by weighing in the views, preferences and experiences of stakeholders [147]. We involved a large number of clinical and community stakeholders in designing this review and developing evidence-based FES cycling clinical practice guidelines (manuscript in preparation). This process demonstrated that many people with SCI and their health-care providers encourage the cautious use of evidence beyond gold-standard RCTs, given the importance they see in deploying FES cycling in clinical and community environments.

A limitation of this review is the use of counting the number of studies showing statistically significant improvements [2, 148]. However, lack of established benchmarks for clinically meaningful improvements in SCI [31], and mere absence of reporting mean differences, effect sizes, 95% confidence intervals, or individual data, rendered this the best possible approach towards synthesizing the evidence [2]. Although this approach increased the risk of type II errors and family-wise error rates [148], it is unlikely that such errors influenced the primary findings of this review, as significant improvements were found in nearly all studies and outcome measures related to muscle health, power output and aerobic fitness.

### Implications for future research: gaps identified in this review

The gaps in the evidence identified in this review can inform the prioritization and direction of future research. It was encouraging to observe that the research base for FES cycling after SCI has steadily increased since the 1980s, with many new studies conducted over the last decade (e.g., almost half of all included studies in this review were published between 2010 and 2020). Important evidence gaps remain however, and clinical practice and policy development would be served by addressing these.

One key gap is the current lack of high-quality evidence on potential functional or neurological benefits of FES cycling. The few Level 3 pre-post studies identified in this review showed some improvements in adults with chronic SCI. Animal studies have suggested that initiating exercise during a critical early period may enhance functional recovery [149]. However, lacking are high-quality FES cycling controlled trials taking place within the first 3–6 months after SCI when recovery is most likely [150], while focusing on underlying mechanisms, and functional and neurological outcomes sensitive to change. Such trials can also inform the ongoing debate about the potential of FES cycling for neurorecovery [24].

Other key research gaps identified by this review relate to potential effects of FES cycling on the risk of cardiometabolic disease [141, 151], reduction of debilitating secondary health conditions such as pressure injuries, chronic pain, and urinary tract infections [152–154], and enhancement of subjective well-being [155, 156]. For these outcomes, the review highlighted a lack of high-quality research employing instruments sensitive to exercise-induced changes in adults with SCI that can provide insight into the magnitude of potential improvement of these outcomes following SCI. Such research should be aligned and combined with what SCI users of FES cycling often report anecdotally, such as functional and neurological improvements and psychological benefits.

Changes in many of these outcomes may require intervention periods over a longer period (e.g., 1–2 years) than what most FES cycling studies have used so far (on average 16 weeks, see Table 3). For example, structural cardiac and vascular improvements may occur secondary to adaptations in muscle health and aerobic fitness, but might not be visible in the first months of a person with SCI engaging in FES or other forms of exercise [157]. If they occur, changes in bone health may require at least one year of FES cycling exercise [87, 158, 159].

RCTs over such long periods are costly, likely face ethical challenges, and are often not feasible due to small potential participant pools [146]. A solution is the use of longitudinal designs taking place in clinical and community centres where FES cycling is used daily as part of ongoing rehabilitation and exercise programs. The statistical power and high external validity of such a design, combined with high-quality reporting of the intervention details and environment, could provide a wealth of information about a range of outcomes on which future clinical practice guidelines can be build. An additional or alternative successful approach could be home-based FES cycling [58], in particular when combined with better user education and establishment of user-specific goals between a practitioner and a person with SCI [3].

The intervention studies identified by this review did not analyse or provide sufficient information to draw conclusions about the minimum or optimal dose of FES cycling exercise and which neuromuscular stimulation characteristics would be required for that. This highlights a need for more robust comparisons of FES exercise prescriptions and approaches to selecting neuromuscular stimulation characteristics, how to keep providing progressive overload for continued improvements, and how to best deal with the “fatigue” problem due to reverse-order muscle fibre recruitment [160–162]. This for example requires novel comparative studies on dose–response and stimulation strategies tailored towards informing clinical practice guideline development.

Finally, current limitations of the evidence base, which prohibited meaningful synthesis of the evidence using forest plots and meta-analysis, could be overcome by improving reporting quality and establishing standardized outcome measures for each outcome category. The relatively poor scores on the risk of bias assessments highlight the need for better description of randomization procedures, intervention protocols, control conditions, dropout rates, sample size calculations, effect sizes, confidence intervals, and incidence of adverse events, in accordance with international reporting standards [163–165]. Using a set of standardized outcome measures would enlarge the potential for a clinically relevant meta-analysis. Provision of data specific for subgroups with different levels of injury and impairment scales could help determine potential differences in effects among various groups of people with SCI.

## Conclusion

The current evidence indicates that FES cycling exercise improves lower-body muscle health (e.g., muscle mass, fiber type composition) of adults with SCI, and may increase power output and aerobic fitness (e.g., peak power and oxygen uptake during an FES cycling test). The evidence summarized and appraised in this review can inform the development of the first international, evidence-based clinical practice guidelines for the use of FES cycling exercise in clinical and community settings of adults with SCI. Ultimately, these clinical practice guidelines help to shape lifelong rehabilitation care plans for the SCI population that fit national and local care contexts and resources.

## Data Availability

All data generated or analyzed during this study are included in this paper or in the online repository at https://osf.io/u9mvx/ including details on data extraction, risk of bias, grey literature search, search strings, and the full reference list of the 97 eligible articles.

## Abbreviations

AIS: American Spinal Injury Association Impairment Scale
BMD: Bone mineral density
GRADE: Grading of Recommendations Assessment, Development and Evaluation
FES: Functional electrical stimulation
RCTs: Randomized controlled trials
SCI: Spinal cord injury
